# (2*RS*)-3-Hydr­oxy-2-methyl-2-(2-pyrid­yl)imidazolidine-4-one

**DOI:** 10.1107/S1600536809030840

**Published:** 2009-08-08

**Authors:** Turganbay S. Iskenderov, Irina A. Golenya, Elźbieta Gumienna-Kontecka, Igor O. Fritsky, Elena V. Prisyazhnaya

**Affiliations:** a Karakalpakian University, Department of Chemistry, Universitet Keshesi 1, 742012 Nukus, Uzbekistan; b Kiev National Taras Shevchenko University, Department of Chemistry, Volodymyrska str. 64, 01601 Kiev, Ukraine; c Faculty of Chemistry, University of Wrocław, 14 F. Joliot-Curie str., 50-383 Wrocław, Poland; d Kyiv National University of Construction and Architecture, Department of Chemistry, Povitroflotsky Ave., 31, 03680 Kiev, Ukraine

## Abstract

The title structure, C_9_H_11_N_3_O_2_, is a racemate. The chiral centre is situated at the N—C—N C atom of the imidazolidine ring. The inter­planar angle between the mean planes of the pyridine and imidazolidine rings is 89.41 (5)°. The methyl group is in a *trans* position with respect to the pyridine N atom. In the crystal, the mol­ecules are arranged in zigzag layers parallel to the *b* axis. The mol­ecules within the layers are inter­connected by strong O—H⋯N and weak N—H⋯O hydrogen bonds; the former take place between OH groups and amine N atoms and the latter between the amine N atom and the carbonyl O atom. In addition, C—H⋯O inter­actions are also present.

## Related literature

For background to hydroxamic acids in biological and coord­ination chemistry, see: Miller (1989 ▶); Lipczynska-Kochany (1991 ▶); Kurzak *et al.* (1992 ▶); Whittaker *et al.* (1999 ▶). For reactions of α-amino hydroxamic acids with aldehydes and ketones resulting in 3-hydroxy­imidazolidin-4-one derivatives, see: Vystorop *et al.* (2002 ▶, 2003 ▶); Marson & Pucci (2004 ▶). For related structures, see: Krämer & Fritsky (2000 ▶); Świątek-Kozłowska *et al.* (2000 ▶); Krämer *et al.* (2002 ▶); Kovbasyuk *et al.* (2004 ▶). For the synthesis, see: Cunningham *et al.* (1949 ▶). For hydrogen bonds, see: Desiraju & Steiner (1999 ▶).
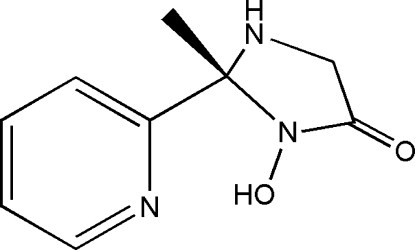


## Experimental

### 

#### Crystal data

C_9_H_11_N_3_O_2_
*M* *_r_* = 193.21
Monoclinic, 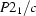

*a* = 8.207 (2) Å
*b* = 10.604 (2) Å
*c* = 10.642 (2) Å
β = 106.43 (3)°
*V* = 888.3 (3) Å^3^
*Z* = 4
Mo *K*α radiation
μ = 0.11 mm^−1^
*T* = 100 K
0.25 × 0.17 × 0.12 mm

#### Data collection

Kuma KM-4-CCD diffractometer
Absorption correction: multi-scan (*CrysAlis RED*, Oxford Diffraction, 2006 ▶) *T*_min_ = 0.976, *T*_max_ = 0.986
6025 measured reflections
2048 independent reflections
1772 reflections with *I* > 2σ(*I*)
*R*_int_ = 0.020

#### Refinement

*R*[*F* ^2^ > 2σ(*F* ^2^)] = 0.039
*wR*(*F* ^2^) = 0.092
*S* = 1.12
2048 reflections
137 parameters
H atoms treated by a mixture of independent and constrained refinement
Δρ_max_ = 0.31 e Å^−3^
Δρ_min_ = −0.21 e Å^−3^

### 

Data collection: *CrysAlis CCD* (Oxford Diffraction, 2006 ▶); cell refinement: *CrysAlis RED* (Oxford Diffraction, 2006 ▶); data reduction: *CrysAlis RED*; program(s) used to solve structure: *SHELXS97* (Sheldrick, 2008 ▶); program(s) used to refine structure: *SHELXL97* (Sheldrick, 2008 ▶); molecular graphics: *ORTEP-3 for Windows* (Farrugia, 1997 ▶); software used to prepare material for publication: *WinGX* (Farrugia, 1999 ▶).

## Supplementary Material

Crystal structure: contains datablocks global, I. DOI: 10.1107/S1600536809030840/fb2163sup1.cif

Structure factors: contains datablocks I. DOI: 10.1107/S1600536809030840/fb2163Isup2.hkl

Additional supplementary materials:  crystallographic information; 3D view; checkCIF report

## Figures and Tables

**Table 1:** Hydrogen-bond geometry (Å, °)

*D*—H⋯*A*	*D*—H	H⋯*A*	*D*⋯*A*	*D*—H⋯*A*
O1—H1*O*⋯N1^i^	0.95 (2)	1.78 (2)	2.7287 (16)	175.5 (18)
N1—H1*N*⋯O2^ii^	0.874 (17)	2.135 (18)	3.0058 (15)	173.7 (15)
C6—H6⋯O1^iii^	0.93	2.47	3.2867 (17)	147
C7—H7⋯O2^iii^	0.93	2.81	3.3559 (17)	119
C8—H8⋯O2^iv^	0.93	2.80	3.4177 (17)	125

Symmetry codes: (i) 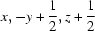; (ii) 
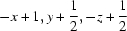
; (iii) 
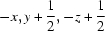
; (iv) 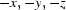.

**Fig. 1.**
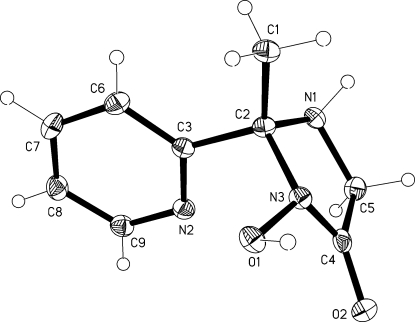
A view of the (2R)-enantiomer of the title compound, with the displacement ellipsoids shown at the 50% probability level.

**Fig. 2.**
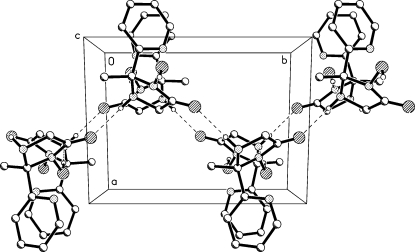
A packing diagram of the title compound. Oxygen and nitrogen atoms are depicted as larger (hatched) and smaller (dotted) circles, respectively. The O-H···N and N-H···O hydrogen bonds are indicated by the dashed lines. The H atoms not involved in the hydrogen bonding have been omitted for the sake of clarity.

## supplementary crystallographic information

### Comment

Hydroxamic acids are important bioligands possessing a wide spectrum of biological activities (Lipczynska-Kochany, 1991). Notably, they have a high affinity to the transition metal ions (Kurzak *et al.*, 1992). For example, naturally occurring hydroxamate siderophores are strong Fe(III) chelators (Miller, 1989). Hydroxamic acids are also efficient metalloenzyme inhibitors, *e.g.* urease and matrix metalloproteinase inhibitors (Whittaker *et al.*, 1999).

These properties have provoked current interest in the development of novel synthetic routes for preparation of new selective hydroxamate chelating agents and siderophore mimics. Recently it was found that the reactions of α-amino hydroxamic acids with aldehydes and ketons do not result in the open-chain Schiff base hydroxamic acids but afford five-membered cyclic products containing residues of 3-hydroxyimidazolidine-4-one (Marson & Pucci, 2004; Vystorop *et al.*, 2002; Vystorop *et al.*, 2003). Here we describe a crystal structure of the title structure, 2-methyl-2-(pyridine-2-yl)-3-hydroxyimidazolidine-4-one, obtained as a result of the condensation of glycine hydroxamic acid and 2-acetylpyridine.

The molecules of the title structure are interconnected by the H-bonds. The molecules contain a chiral centre at the C2 atom (Fig. 1) and the structure is a racemate. The molecule is not planar: the interplanar angle between the mean planes of the pyridine and imidazolidine rings equals to 89.41 (5)°. The imidazolidine ring exhibits the envelope conformation: the C2 atom is displaced by 0.320 (2) Å out of the mean plane defined by four other atoms of the ring. The methyl group is in the *trans*-position with respect to the pyridine nitrogen.

The bond lengths C—O, N—O and C—N in the hydroxamic function suggest the presence of the hydroxamic function in the hydroxamic form rather than in the oximic one (Świątek-Kozłowska *et al.*, 2000). The C—N and C—C bond lengths within the pyridine ring are normal for 2-substituted pyridine derivatives (Krämer & Fritsky, 2000; Krämer *et al.*, 2002; Kovbasyuk *et al.*, 2004).

In the crystal packing, the molecules are arranged into zig-zagged layers by the O1—H···N2 and N2—H···O2 hydrogen bonds. These layers are parallel to the axis *b*. The former one takes place between NOH group and it is considered as a strong hydrogen bond (Desiraju & Steiner, 1999) while the latter one between the amine nitrogen and the carbonyl oxygen atom (Fig. 2) is considered as weak one (Desiraju & Steiner, 1999). Moreover, the mentioned layers are interconnected by C—H···O H-bonds (Tab. 1).

### Experimental

A suspension of glycine hydroxamic acid (0.9 g, 10 mmol) and 2-acetylpyridine (12 mmol) in 30 ml of 96% aqueous ethanol was refluxed for at 78°C for 1-2 h. The hot reaction mixture was filtered, the filtrate produced a white precipitate on cooling. The precipitate was filtered, air-dried and recrystallized from absolute ethanol to yield the title structure as colourless prismatic crystals of average size 0.25 × 0.15 × 0.15 mm. The reagent, glycine hydroxamic acid, was prepared according to the procedure described by Cunningham *et al.* (1949).

### Refinement

All the H-atoms were discernible in the difference electron density map. The coordinates and the isotropic displacement parameters of the hydroxyl and amine hydrogens that are involved in the strongest hydrogen bonds have been refined. The hydrogens with C atoms as their carriers were situated into the idealized positions and constrained: C—H = 0.93, 0.96 and 0.97 Å for aryl, methyl and methylene hydrogens; U_iso_H_aryl/methylene_=1.2U_eq_C_aryl/methylene_, U_iso_H_methyl_=1.5U_eq_C_methyl_. The methyl H atoms have been refined with AFIX 137 [*SHELXL98* (Sheldrick, 2008] so their positions with regard to the electron density maps have been optimized.

### Crystal data

**Table:**

C_9_H_11_N_3_O_2_	*F*(000) = 408
*M**_r_* = 193.21	*D*_x_ = 1.445 Mg m^−^^3^
Monoclinic, *P*2_1_/*c*	Mo *K*α radiation, λ = 0.71073 Å
Hall symbol: -P 2ybc	Cell parameters from 505 reflections
*a* = 8.207 (2) Å	θ = 4.5–27.0°
*b* = 10.604 (2) Å	µ = 0.11 mm^−^^1^
*c* = 10.642 (2) Å	*T* = 100 K
β = 106.43 (3)°	Block, colourless
*V* = 888.3 (3) Å^3^	0.25 × 0.17 × 0.12 mm
*Z* = 4	

### Data collection

**Table:**

Kuma KM-4-CCD diffractometer	2048 independent reflections
Radiation source: fine-focus sealed tube	1772 reflections with *I* > 2σ(*I*)
graphite	*R*_int_ = 0.020
ω scans	θ_max_ = 28.4°, θ_min_ = 3.4°
Absorption correction: multi-scan (*CrysAlis RED*, Oxford Diffraction, 2006)	*h* = −10→10
*T*_min_ = 0.976, *T*_max_ = 0.986	*k* = −12→14
6025 measured reflections	*l* = −10→13

### Refinement

**Table:**

Refinement on *F*^2^	Secondary atom site location: difference Fourier map
Least-squares matrix: full	Hydrogen site location: difference Fourier map
*R*[*F*^2^ > 2σ(*F*^2^)] = 0.039	H atoms treated by a mixture of independent and constrained refinement
*wR*(*F*^2^) = 0.092	*w* = 1/[σ^2^(*F*_o_^2^) + (0.0423*P*)^2^ + 0.2114*P*] where *P* = (*F*_o_^2^ + 2*F*_c_^2^)/3
*S* = 1.12	(Δ/σ)_max_ < 0.001
2048 reflections	Δρ_max_ = 0.31 e Å^−^^3^
137 parameters	Δρ_min_ = −0.21 e Å^−^^3^
0 restraints	Extinction correction: *SHELXL97* (Sheldrick, 2008), Fc^*^=kFc[1+0.001xFc^2^λ^3^/sin(2θ)]^-1/4^
35 constraints	Extinction coefficient: 0.011 (3)
Primary atom site location: structure-invariant direct methods	

### Special details

**Table:**

Geometry. All e.s.d.'s (except the e.s.d. in the dihedral angle between two l.s. planes) are estimated using the full covariance matrix. The cell e.s.d.'s are taken into account individually in the estimation of e.s.d.'s in distances, angles and torsion angles; correlations between e.s.d.'s in cell parameters are only used when they are defined by crystal symmetry. An approximate (isotropic) treatment of cell e.s.d.'s is used for estimating e.s.d.'s involving l.s. planes.
Refinement. Refinement of *F*^2^ against ALL reflections. The weighted *R*-factor *wR* and goodness of fit *S* are based on *F*^2^, conventional *R*-factors *R* are based on *F*, with *F* set to zero for negative *F*^2^. The threshold expression of *F*^2^ > σ(*F*^2^) is used only for calculating *R*-factors(gt) *etc*. and is not relevant to the choice of reflections for refinement. *R*-factors based on *F*^2^ are statistically about twice as large as those based on *F*, and *R*- factors based on ALL data will be even larger.

### Fractional atomic coordinates and isotropic or equivalent isotropic displacement parameters (Å^2^)

**Table:**

	*x*	*y*	*z*	*U*_iso_*/*U*_eq_	
O1	0.16520 (11)	0.16310 (8)	0.36523 (9)	0.0161 (2)	
O2	0.40231 (11)	0.01414 (8)	0.28974 (9)	0.0197 (2)	
N1	0.33317 (13)	0.31162 (10)	0.12474 (10)	0.0143 (2)	
N2	0.02391 (13)	0.16898 (10)	0.05476 (10)	0.0162 (2)	
N3	0.27351 (13)	0.20590 (10)	0.29470 (10)	0.0135 (2)	
C1	0.21832 (17)	0.43456 (12)	0.27885 (13)	0.0175 (3)	
H1A	0.1455	0.4293	0.3351	0.026*	
H1B	0.1815	0.5026	0.2179	0.026*	
H1C	0.3330	0.4496	0.3307	0.026*	
C2	0.21024 (15)	0.31194 (11)	0.20447 (11)	0.0137 (3)	
C3	0.03258 (15)	0.27995 (11)	0.11645 (11)	0.0137 (3)	
C4	0.36622 (15)	0.12145 (12)	0.24864 (12)	0.0147 (3)	
C5	0.41829 (16)	0.18664 (12)	0.14047 (12)	0.0167 (3)	
H5A	0.5407	0.1966	0.1638	0.020*	
H5B	0.3818	0.1385	0.0598	0.020*	
C6	−0.10579 (17)	0.36021 (12)	0.09906 (13)	0.0184 (3)	
H6	−0.0958	0.4360	0.1446	0.022*	
C7	−0.25965 (17)	0.32455 (13)	0.01199 (13)	0.0206 (3)	
H7	−0.3546	0.3762	−0.0018	0.025*	
C8	−0.26922 (16)	0.21144 (13)	−0.05353 (13)	0.0196 (3)	
H8	−0.3702	0.1859	−0.1130	0.023*	
C9	−0.12508 (16)	0.13664 (12)	−0.02886 (12)	0.0177 (3)	
H9	−0.1323	0.0601	−0.0727	0.021*	
H1O	0.229 (3)	0.1711 (18)	0.454 (2)	0.049 (6)*	
H1N	0.409 (2)	0.3703 (16)	0.1556 (15)	0.023 (4)*	

### Atomic displacement parameters (Å^2^)

**Table:**

	*U*^11^	*U*^22^	*U*^33^	*U*^12^	*U*^13^	*U*^23^
O1	0.0174 (5)	0.0199 (5)	0.0126 (4)	−0.0039 (3)	0.0067 (4)	0.0010 (3)
O2	0.0196 (5)	0.0154 (5)	0.0233 (5)	0.0026 (3)	0.0050 (4)	0.0021 (4)
N1	0.0146 (5)	0.0152 (5)	0.0135 (5)	−0.0028 (4)	0.0049 (4)	−0.0007 (4)
N2	0.0169 (5)	0.0145 (5)	0.0166 (5)	−0.0009 (4)	0.0040 (4)	−0.0016 (4)
N3	0.0147 (5)	0.0148 (5)	0.0117 (5)	−0.0010 (4)	0.0052 (4)	0.0016 (4)
C1	0.0208 (7)	0.0143 (6)	0.0172 (6)	−0.0012 (5)	0.0052 (5)	−0.0020 (5)
C2	0.0165 (6)	0.0132 (6)	0.0118 (6)	0.0003 (4)	0.0046 (5)	0.0010 (4)
C3	0.0162 (6)	0.0139 (6)	0.0117 (6)	−0.0012 (4)	0.0050 (5)	0.0011 (4)
C4	0.0111 (6)	0.0170 (6)	0.0139 (6)	−0.0022 (4)	−0.0001 (4)	−0.0033 (5)
C5	0.0161 (6)	0.0186 (6)	0.0160 (6)	0.0012 (5)	0.0057 (5)	−0.0010 (5)
C6	0.0211 (7)	0.0155 (6)	0.0187 (6)	0.0025 (5)	0.0060 (5)	−0.0019 (5)
C7	0.0173 (7)	0.0221 (7)	0.0216 (7)	0.0063 (5)	0.0042 (5)	0.0027 (5)
C8	0.0156 (6)	0.0236 (7)	0.0171 (6)	−0.0009 (5)	0.0008 (5)	0.0017 (5)
C9	0.0194 (7)	0.0161 (6)	0.0167 (6)	−0.0022 (5)	0.0036 (5)	−0.0029 (5)

### Geometric parameters (Å, °)

**Table:**

O1—N3	1.3917 (13)	C1—H1C	0.9600
O1—H1O	0.95 (2)	C2—C3	1.5316 (18)
O2—C4	1.2250 (16)	C3—C6	1.3891 (17)
N1—C5	1.4854 (16)	C4—C5	1.5047 (17)
N1—C2	1.4907 (16)	C5—H5A	0.9700
N1—H1N	0.874 (17)	C5—H5B	0.9700
N2—C9	1.3376 (17)	C6—C7	1.3908 (19)
N2—C3	1.3395 (16)	C6—H6	0.9300
N3—C4	1.3536 (16)	C7—C8	1.3783 (19)
N3—C2	1.4744 (16)	C7—H7	0.9300
C1—C2	1.5139 (17)	C8—C9	1.3863 (19)
C1—H1A	0.9600	C8—H8	0.9300
C1—H1B	0.9600	C9—H9	0.9300
			
N3—O1—H1O	104.8 (12)	C6—C3—C2	123.14 (11)
C5—N1—C2	108.08 (9)	O2—C4—N3	126.10 (12)
C5—N1—H1N	109.4 (11)	O2—C4—C5	127.42 (11)
C2—N1—H1N	107.8 (10)	N3—C4—C5	106.47 (11)
C9—N2—C3	117.59 (11)	N1—C5—C4	105.65 (10)
C4—N3—O1	119.32 (10)	N1—C5—H5A	110.6
C4—N3—C2	113.53 (10)	C4—C5—H5A	110.6
O1—N3—C2	116.04 (9)	N1—C5—H5B	110.6
C2—C1—H1A	109.5	C4—C5—H5B	110.6
C2—C1—H1B	109.5	H5A—C5—H5B	108.7
H1A—C1—H1B	109.5	C3—C6—C7	118.43 (12)
C2—C1—H1C	109.5	C3—C6—H6	120.8
H1A—C1—H1C	109.5	C7—C6—H6	120.8
H1B—C1—H1C	109.5	C8—C7—C6	119.00 (12)
N3—C2—N1	101.45 (9)	C8—C7—H7	120.5
N3—C2—C1	111.05 (10)	C6—C7—H7	120.5
N1—C2—C1	111.28 (10)	C7—C8—C9	118.60 (12)
N3—C2—C3	109.17 (10)	C7—C8—H8	120.7
N1—C2—C3	109.38 (9)	C9—C8—H8	120.7
C1—C2—C3	113.79 (10)	N2—C9—C8	123.35 (12)
N2—C3—C6	123.02 (12)	N2—C9—H9	118.3
N2—C3—C2	113.82 (10)	C8—C9—H9	118.3
			
C4—N3—C2—N1	−23.00 (12)	N1—C2—C3—C6	−120.73 (13)
O1—N3—C2—N1	−166.82 (9)	C1—C2—C3—C6	4.41 (17)
C4—N3—C2—C1	−141.35 (11)	O1—N3—C4—O2	−21.10 (17)
O1—N3—C2—C1	74.84 (13)	C2—N3—C4—O2	−163.63 (11)
C4—N3—C2—C3	92.40 (12)	O1—N3—C4—C5	160.17 (9)
O1—N3—C2—C3	−51.42 (13)	C2—N3—C4—C5	17.65 (13)
C5—N1—C2—N3	18.56 (12)	C2—N1—C5—C4	−9.58 (12)
C5—N1—C2—C1	136.75 (10)	O2—C4—C5—N1	176.85 (11)
C5—N1—C2—C3	−96.68 (11)	N3—C4—C5—N1	−4.45 (13)
C9—N2—C3—C6	1.26 (18)	N2—C3—C6—C7	−1.06 (19)
C9—N2—C3—C2	−176.93 (11)	C2—C3—C6—C7	176.96 (11)
N3—C2—C3—N2	−52.74 (13)	C3—C6—C7—C8	0.01 (19)
N1—C2—C3—N2	57.46 (13)	C6—C7—C8—C9	0.7 (2)
C1—C2—C3—N2	−177.41 (10)	C3—N2—C9—C8	−0.43 (19)
N3—C2—C3—C6	129.08 (12)	C7—C8—C9—N2	−0.6 (2)

### Hydrogen-bond geometry (Å, °)

**Table:**

*D*—H···*A*	*D*—H	H···*A*	*D*···*A*	*D*—H···*A*
O1—H1O···N1^i^	0.95 (2)	1.78 (2)	2.7287 (16)	175.5 (18)
N1—H1N···O2^ii^	0.874 (17)	2.135 (18)	3.0058 (15)	173.7 (15)
C6—H6···O1^iii^	0.93	2.47	3.2867 (17)	147
C7—H7···O2^iii^	0.93	2.81	3.3559 (17)	119
C8—H8···O2^iv^	0.93	2.80	3.4177 (17)	125

Symmetry codes: (i) *x*, −*y*+1/2, *z*+1/2; (ii) −*x*+1, *y*+1/2, −*z*+1/2; (iii) −*x*, *y*+1/2, −*z*+1/2; (iv) −*x*, −*y*, −*z*.
